# Harnessing melanin as a marine natural product: a new perspective on food industry applications

**DOI:** 10.7717/peerj.21855

**Published:** 2026-11-10

**Authors:** Ain Farina Farizan, Mohd Amran Aaqillah-Amr, Kamariah Bakar, Mohd Al-Hafiz Zaini, Nor-Khaizura Mahmud, Poh Wai Chia, Najiah Musa, Nurul Ulfah Karim

**Affiliations:** 1 Higher Institution Centre of Excellence (HICoE), Institute of Tropical Aquaculture and Fisheries, Universiti Malaysia Terengganu, Kuala Nerus, Terengganu, Malaysia; 2 Higher Institution Centre of Excellence (HICoE), Institute of Climate Adaptation and Marine Biotechnology, Universiti Malaysia Terengganu, Kuala Nerus, Terengganu, Malaysia; 3 Mega Seafood Sdn Bhd, Ulu Tiram, Johor, Malaysia; 4 Faculty of Food Science and Technology, Universiti Putra Malaysia, Serdang, Selangor, Malaysia; 5 Institute of Tropical Agriculture and Food Security, Universiti Putra Malaysia, Serdang, Selangor, Malaysia; 6 Faculty of Science and Marine Environment, Universiti Malaysia Terengganu, Kuala Nerus, Terengganu, Malaysia; 7 Faculty of Fisheries and Aquaculture Sciences, Universiti Malaysia Terengganu, Kuala Nerus, Terengganu, Malaysia

**Keywords:** Melanin, Allomelanin, Eumelanin, Neuromelanin, Pheomelanin, Food

## Abstract

Derived from the Greek word “melanos”, melanin is a biopolymer that can be extracted from various sources, including animals, plants, and microorganisms. Four types of melanin have been identified based on their color and synthesis pathway: (i) dark-brown to black allomelanin, (ii) black-brown eumelanin, (iii) dark-brown neuromelanin, and (iv) yellow to reddish-brown pheomelanin. Melanin is widely recognized as a photoprotective, antimicrobial, and antioxidant agent, and has gained increasing attention for its potential applications in the pharmaceutical, cosmetic, environmental remediation , and food industries. The growing demand for healthier food products in the modern food industry has driven research and development in areas such as nutraceutical food ingredients, advanced packaging technologies, natural-based additives, and improved production technologies . Although several reviews have discussed various aspects of melanin, studies specifically focusing on melanin derived from animal sources and its applications in the food industry remain limited. Therefore, this review aims to summarize the extraction methods of melanin from animal sources and discuss its potential functions and applications in the food industry. These insights provide guidance for researchers in developing sustainable extraction and application strategies, support the industry in replacing synthetic additives with natural alternatives, and inform consumers about functional, health-promoting food innovations.

## Introduction

Food includes raw, semi-processed, and fully processed consumable products deliberately taken by humans and plays a significant role in providing essential nutrition for physiological functions, growth, and energy. The United Nations (UN) predicted that by 2050 the global human population will increase to 9.7 billion people. This increase in population will lead to an increase in the food industry production rate in order to meet consumer needs (Hamad & Tayel, in press). Therefore, improved operations and management in the food manufacturing sector are required, covering processes from preparing agricultural raw materials to turning them into food products that fulfil the nutritional needs of consumers (Canatan et al., 2025). All these food preparation steps are correlated to economic expansion, human health, sustainable development, and green living (Michel et al., 2024) which encourage the exploration of natural bioactive compounds that can improve food quality, safety, and functionality.

Melanin is a natural pigment that gives vibrant appearance and color diversity to many organisms, including humans, animals, plants, and microorganisms (Alam et al., 2022). Melanin has received increasing research attention in a wide range of industrial applications, including food manufacturing (Yang et al., 2023), agriculture (Muñoz Torres, Cárdenas-Ninasivincha & Aguilar, 2024), environmental remediation (Oh et al., 2021), biomedical applications (Caldas et al., 2020), pharmaceutical production (Cheng et al., 2025), and cosmetology (Menichetti et al., 2024). This rapid diversification reflects the growing interest in melanin as a multifunctional biopolymer with potential across multiple technological domains. Its primary uses are attributed to its distinctive physicochemical properties, such as metal-chelating capacity, antioxidant activity, free radical scavenging ability, and strong UV absorption (Tong et al., 2023). Covering a colour range from brown to black, melanin has a broad spectrum in terms of light absorption, which enables absorption of visible, ultraviolet, and infrared radiation. These properties help to prevent deterioration of living tissues and protect cellular DNA from mutations (Singh et al., 2025a). Besides that, the antioxidant activity of melanin is contributed by its electron acceptor and donor nature, known as redox behaviour. Its ability to participate in redox reactions allows the neutralization of free radicals and reactive oxygen species (ROS) to occur (Menichetti et al., 2025). Other than that, melanin also plays a role as an immunological defence agent that can bind with metals, toxins, microorganisms, and drugs thereby reducing their harmful effects on the body (Madkhali & Algessair, 2024).

Despite the wide availability of natural pigments and functional compounds, many existing food additives suffer from poor thermal stability, limited antioxidant capacity, and safety concerns associated with synthetic alternatives (Silva, Reboredo & Lidon, 2022). In this context, natural melanin presents a unique advantage due to its multifunctional properties, including colourant capability, antioxidant, antimicrobial, and photoprotective functions. Its high stability under heat, light, and pH variations further supports its suitability for food applications. At the same time, previous reviews have addressed melanin extraction, chemical structure, and general applications (Alam et al., 2022; Guo et al., 2023; Pralea et al., 2019), however, limited attention has been given to melanin derived from marine animal sources in food systems. Therefore, exploring melanin not only addresses consumer demand for clean-label ingredients but also supports the development of sustainable and functional food products. By integrating evidence from prior studies and highlighting remaining research gaps, this review aims to guide researchers, inform industry stakeholders, and provide the general public with insights into the potential benefits of natural melanin as a functional, safe, and sustainable food ingredient.

The journal is intended to serve academicians, scholars, and industry practitioners dealing with food science and food technology focusing on the use of natural materials, especially melanin, in producing food colouring, additives and flavour enhancers, functional food ingredients, and various types of food packaging. This journal also covers the functions of natural melanin, melanin-producing organisms, and several melanin extraction methods. This review highlights the potential and health benefits of natural melanin as an alternative nutritional component and as a replacement for chemically synthesized food additives.

## Survey Methodology

In order to identify related and relevant literature, we conducted a comprehensive search using the keywords ‘animal melanin’, ‘extraction of melanin’, ‘melanin in food industry’ and ‘squid ink melanin’ in the Google Scholar (https://scholar.google.com), PubMed (https://pubmed.ncbi.nlm.nih.gov), and Science Direct (https://www.sciencedirect.com) databases. In this review, both vertebrate and invertebrate animals reported to potentially produce melanin from 2010 to 2025 are discussed. Key data, including definitions, objectives, extraction techniques, and applications were collected, organized into appropriate categories, and all selected articles were included using an unbiased approach.

## Natural Melanin

Natural melanin is classified into four types: allomelanin, eumelanin, neuromelanin, and pheomelanin (Guo et al., 2023). These types are found in both vertebrates and invertebrates, where they perform various biological functions. Allomelanin is a non-nitrogenous pigment, typically dark brown to black, commonly found in fungi, where it helps protect against harsh environmental conditions (Zhou et al., 2019). Eumelanin is widely present in hair, eyes, and skin and is known for its protective role against UV radiation and its metal-chelating properties (Xie et al., 2019). Due to these properties, it is the most studied type of melanin. Neuromelanin is a dark pigment found in specific neurons in the brain, particularly in the substantia nigra, where it helps remove toxic molecules and metal ions (Sacchini et al., 2022; Capucciati et al., 2021). Pheomelanin is a sulfur-containing pigment responsible for yellow to reddish-brown coloration, commonly associated with red hair and light skin, and also found in some animals (Kurkiewicz et al., 2025). A summary of melanin distribution, types, and functions across vertebrate and invertebrate organisms is presented in Table 1, highlighting its diverse biological roles.

### Melanin in vertebrates

In aquatic species such as fish, melanin is produced mainly by melanophores in the dermis, where it contributes to body coloration and adaptive camouflage. It also provides functional protection, particularly in the eyes, where eumelanin helps shield tissues from UV radiation and environmental stressors. Variations in pigmentation intensity have been linked to environmental conditions such as light exposure, which influence melanin expression and resulting body coloration (Bjørgen & Koppang, 2024).

**Table 1:** Melanin distribution, types, and functional roles in vertebrate and invertebrate organisms.

**Category**	**Species (Scientific name)**	**Melanin type**	**Location in organism**	**Key function**	**Author(s)**
Vertebrates	Alpaca (*Vicugna pacos*)	Pheomelanin	Fleece	Pigmentation contributing to fleece coloration and commercial value	Cransberg, Wakamatsu & Munyard (2013)
	Passerine birds (*Phoenicurus phoenicurus*, *Sylvia atricapilla*)	Eumelanin, pheomelanin	Feathers (plumage)	Coloration, visual signaling, metal ion binding, and detoxification	Zduniak et al. (2014)
	Barn Swallows (*Hirundo rustica erythrogaster*)	Eumelanin, pheomelanin	Feathers (plumage)	Melanin composition influences plumage coloration and sex-related variation	McGraw, Safran & Wakamatsu (2005)
	Chinese softshell turtle (*Pelodiscus sinensis*)	Eumelanin, pheomelanin	Skin	Skin coloration determined by differential melanin distribution	Qi et al. (2024)
	Human (*Homo sapiens*)	Eumelanin, pheomelanin	Hair	Variations in eumelanin content across hair colors, with distinct pheomelanin presence in red hair.	Ito et al. (2011)
	Human (*Homo sapiens*)	Neuromelanin	Substantia nigra	Metal chelation and neuroprotection in dopaminergic neurons	Double et al. (2003); Zecca et al. (2008)
	Macaque (*Macaca fascicularis*)	Neuromelanin	Substantia nigra	Neuromelanin accumulation induces *α*-synuclein aggregation and neurodegeneration	Chocarro et al. (2023)
	Mouse (*Mus musculus*)	Eumelanin, pheomelanin	Skin	Regulation of pigmentation *via* melanocortin signaling and tyrosinase pathway	Slominski et al. (2005)
Invertebrates	Insect (*Drosophila melanogaster*)	Eumelanin	Hemocyte	Immune defense *via* melanization response against pathogens	Kohler et al. (2007)
	Spiders (Theraphosidae, Araneidae, Salticidae, Theridiidae)	Eumelanin	Hair, non-black cuticle	Contributes to cuticular pigmentation and optical properties	Hsiung, Blackledge & Shawkey (2015)
	Damselfly (*Coenagrion puella*)	Eumelanin	Cuticle	UV-induced melanization associated with developmental plasticity	Debecker et al. (2015)
	Grasshopper (*Sphingonotus azurescens*)	Pheomelanin	Cuticle	Evidence of sulfur-containing melanin pathway	Galván et al. (2015)
	Molluscs (Bivalvia, Cephalopoda, and Gastropoda)	Eumelanin, pheomelanin	Shell	Shell pigmentation and pattern formation; variable occurrence across taxa	Affenzeller et al. (2019)

**Notes.**

Allomelanin is primarily associated with fungal and plant systems and has not been consistently confirmed in vertebrate or invertebrate animals; therefore, it is excluded from this table.

Across terrestrial vertebrates, birds represent one of the most well-studied groups in terms of melanin diversity. Two main forms, eumelanin and pheomelanin, are responsible for a wide range of coloration patterns from black and brown to yellow and reddish tones (Galván & Solano, 2016; Van Grouw, 2017). Aside from pigmentation, melanin also contributes to protection and biological signaling functions. In some species, melanin is distributed not only in feathers but also in tissues such as skin, bones, and internal organs, where it has been associated with antioxidant and physiological benefits (Chen et al., 2024; Roulin et al., 2024).

In amphibians, melanin is present in specialized chromatophores and contributes to dark coloration patterns that are influenced by environmental conditions. Higher or lower melanin levels can reflect adaptation to temperature and UV exposure, supporting protection of internal tissues from radiation-induced damage (Franco-Belussi, Provete & De Oliveira, 2017; Fu et al., 2022). Both eumelanin and pheomelanin have been reported in amphibian skin and developmental stages, further demonstrating their functional diversity (Wolnicka-Glubisz et al., 2012). In mammals including humans, melanin is primarily responsible for hair and skin pigmentation and also contributes to biological protection. Differences in melanin density and structure have been observed across populations, influencing pigmentation intensity and biological properties. Melanin extracted from hair has shown functional properties including antimicrobial and redox activity (Roldan-Kalil et al., 2023; Kojima et al., 2024).

### Melanin in invertebrates

In invertebrates, melanin is widely distributed across various taxa, including mollusks, cephalopods, arthropods, cnidarians, and echinoderms. In mollusks such as gastropods and bivalves, shell pigmentation has been extensively studied. Eumelanin and pheomelanin are the main pigment types, as reported in the scallop *Patinopecten yessoensis*, which contains approximately 23.4% pheomelanin, with eumelanin as the dominant pigment (Sun et al., 2017). The presence of eumelanin has also been observed in other species through dark shell markings, such as in *Clanculus*, *Mytilus edulis*, and *Nautilus pompilius* (Affenzeller et al., 2019). Cephalopods, including squid and cuttlefish, are among the most studied sources of melanin. Their ink sacs contain about 15% melanin, which functions mainly as a defense mechanism by creating a visual barrier to escape predators (Tamilarasan & Ramasubramaniam, 2019). Eumelanin has been successfully extracted from several species, including *Loligo sp.*, *L. duvauceli* (Sukmiwati, Edison & Nuzul, 2023), *Sepia officinalis* (Caldas et al., 2024), *S. pharaonis* (Le et al., 2024), and *S. esculenta* (Xie et al., 2021). Due to its availability as a seafood by-product and established culinary use, cephalopod ink is considered a promising source of melanin for food applications.

Melanin is also found in insects and other arthropods, where it plays roles in coloration, immunity, and wound healing (Whitten & Coates, 2017). In addition, melanin has been extracted from yeast isolated from the gut of moth *Bombyx mori* (Barretto & Vootla, 2020). In cnidarians, including jellyfish and corals, melanin acts as a protective pigment. It has been identified in the eye cups of *Tripedalia cystophora* and plays roles in stress response, wound healing, and coral bleaching (Parisi et al., 2021). Cnidarians also use melanin as a defense against pathogens, including diseases such as black band disease in corals (Buren et al., 2024).

In echinoderms, such as sea urchins and sea cucumbers, melanin contributes to immune defense and body coloration. It has been detected in sea urchin amoebocytes, where it helps protect against pathogens (Calestani & Wessel, 2018; Li et al., 2021). In sea cucumber (*Apostichopus japonicus*), darker color morphs (dark-green and purple) contain higher melanin levels than lighter ones, indicating a positive relationship between coloration and melanin production (Xing et al., 2017). Overall, invertebrate-derived melanins, particularly from marine organisms, show strong potential for food applications due to their availability, scalability, and compatibility with existing food processing systems.

## Extraction of Melanin

The extraction techniques and yields of melanin vary depending on the source organism and tissue type, which creates challenges in standardizing melanin quality for food-related applications. In animals, melanin extraction typically involves the removal of proteins and other cellular components through treatment with alkaline solutions or enzymes (Wakamatsu & Ito, 2002). Isolating pure melanin can be challenging due to its strong affinity for binding tightly to other cellular constituents. The appropriate extraction and purification methods are largely influenced by the melanin sources, the site of accumulation and the ultimate objective of the study (Pralea et al., 2019). Several extraction methods have been developed to overcome these challenges, including alkaline-acid extraction, enzyme-assisted extraction, ionic-liquids extraction, and water-based extraction. Table 2 summarizes representative studies using these methods across various biological sources under different extraction conditions.

**Table 2:** Summary of reported melanin extraction methods from various biological sources.

**Extraction method**	**Melanin source**	**Solvents**	**Operating conditions**	**Advantages**	**Yield**	**Author(s)**
Alkaline-acid	Human skin and hair	KOH, HCl	Boiling; base-acid cycles	Early method; effective protein removal	∼1.9% from hair; ∼3.08–3.78% from epidermis	Abel & Davis (1896)
	Human hair	NaOH, HCl, ethanol, ether	Repeated base–acid cycles; washing; drying	Thorough removal of proteins/lipids	∼4.8%	Liu et al. (2003)
	Silky fowl (*Gallus gallus*) skin and foot	NH_4_OH, HCl, chloroform, ethyl acetate, ethanol	Alkali extraction; acid precipitation; solvent washing	Antioxidant UV protective properties	n/a	Hsieh & Lien (2012)
	Mutant breed chicken (*Gallus gallus domesticus*) tissue	NaOH, HCl	High-temperature alkali; acid precipitation	Suitable for animal tissue	n/a	Yoo et al. (2017)
	Squid (*Todarodes pacificus*) ink	NaOH, HCl	Mild heating; acid precipitation; purification	Common marine extraction method	n/a	Liu et al. (2023)
	Black soldier fly (*Hermetia illucens*) pupal exuviae	Lactic acid, NaOH, HCl	Demineralize; deproteinate; extraction	Produces water-soluble melanin	n/a	Ambrico et al. (2024)
Enzyme-assisted	Human hair	Enzymes, buffer	Enzymatic digestion at 37 °C	Preserves melanin structure	∼2.3%	Liu et al. (2003)
	Silky fowl (*Gallus gallus domesticus Brisson*) tissues	Buffer, surfactant	Enzymatic + centrifugation purification	Highly purity melanin	up to 21.3 mg/g	Chen et al. (2008)
Ionic-liquids	Alpaca wool	BMIMAc, ethanol	High-temperature dissolution	Retains structure; recyclable solvent	2.2%	Liang et al. (2018)
	Human hair	BMIMCl, H_2_SO_4_	Dissolution + acid precipitation	Preserves structure	6.02%	Luo, Liu & Chen (2023)
	Human hair	BMIM[Cl], HCl	Dissolution + washing + drying	Low environmental impact	16.9%	Mukherjee et al. (2023)
Water	Cuttlefish (*Sepia esculenta*) ink	Water	Homogenization; centrifugation	Simple; preserves nanoparticles	n/a	Wang & Rhim (2019)
	Cuttlefish (*Sepia esculenta*) ink	Water	Dilution; stirring; repeated centrifugation	High yield; preserves stability	87.1%	Xie et al. (2021)

### Alkaline-acid extraction

Both intracellular and extracellular melanin extraction involve alkaline extraction followed by acid precipitation. The alkaline-acid method is commonly employed with modifications tailored to the specific sample type (Pralea et al., 2019; Kurian, 2024). It is a usual practice to break open the outer tissues and use an alkaline solution such as sodium hydroxide to extract melanin. In order to further purify the melanin, a precipitation process with acids such as acetic acid and hydrochloric acid is performed (Kurian, 2024). To achieve higher melanin purity, the crude extract is redissolved in an alkaline solution and subjected to repeated precipitation. At the final stage, the collected precipitate is washed using chloroform and ethanol successively in order to purify the precipitate (Dossou et al., 2022). Even though this method has been widely used in melanin extraction, there are a few drawbacks that need to be considered such as low percentage recovery of the final product, higher production cost, and the use of acidic solution which can cause deterioration of some compounds due to its harsh nature (Choi, 2021; Ghadge et al., 2021a; Tufail et al., 2024).

### Enzyme assisted extraction

Enzyme-assisted extraction offers a green chemistry approach for extracting melanin, particularly from animal keratin fibers and microbial sources. This method utilizes hydrolytic enzymes such as cellulases, pectinases, hemicellulases, and papain to break down cell walls, thereby enhancing solvent penetration and significantly improving melanin recovery. Key factors influencing the efficiency of this method include pH, extraction duration, particle size, and incubation temperature (Łubek-Nguyen, Ziemichód & Olech, 2022). This procedure starts with treating the sample using desired enzymes in a buffer solution followed by incubation for a specified period (Kurian, 2024). After the enzymatic process, the solution undergoes centrifugation to obtain the melanin precipitate (Zhang et al., 2021). Compared to alkaline-acid extraction, the enzymatically extracted melanin preserves the morphology of the melanosomes and is most preferable for extracting human melanin (Pralea et al., 2019). In addition, enzyme-assisted extraction complies with sustainability goals and can produce materials suitable for food, pharmaceutical and cosmetic use.

### Ionic-liquids extraction

Ionic-liquids (ILs) are low temperature liquid organic salts that are commonly based on imidazolium or ammonium compounds. These are considered green solvents and have been widely applied in various fields. ILs can efficiently modify the redox properties of melanin polymers and their solubility as they are capable of dissolving a broad range of cellular materials and biomolecules. Compared to traditional volatile organic solvents, ILs are more environmentally friendly due to their unique properties, including high chemical and thermal stability, non-flammability and low vapor pressure (Ling, Lio & Zhu, 2021; Ambrico et al., 2024). The extraction process begins with heating the sample in ILs at an elevated temperature until it is completely dissolved, followed by centrifugation to separate and collect the precipitate (Liang et al., 2018; Luo, Liu & Chen, 2023). Following the extraction process, the supernatant containing ILs can be recovered through a liquid-liquid extraction method using chloroform. The remaining solvent is then removed by a rotary evaporator (Ghadge et al., 2021b).

### Water extraction

Water-based extraction of melanin is widely used and is considered one of the simplest, safest, and most cost-effective methods. This process involves diluting melanin-containing samples with water, followed by centrifugation to separate the precipitate. Cephalopod ink sacs are among the commonly used samples for this extraction technique. In this technique, the sample material is suspended in distilled water with continuous stirring to promote the release of pigment without involving any harsh chemicals in order to maintain the structure and properties of melanin (Zhou et al., 2019). Water soluble impurities in the supernatant are removed, and the remaining pellet needs to be washed several times in order to obtain a pure melanin sample (Guo et al., 2013). The percentage recovery of melanin extracted *via* this method was reported to be the highest compared to enzyme-assisted and alkaline-acid extraction (Xie et al., 2021). However, this extraction technique can sometimes produce lower yields as it is not able to degrade protein barriers that protect the melanin granule (Thaira et al., 2019).

### Critical evaluation of extraction techniques

Despite the development of various extraction techniques, no single technique achieves an ideal balance across all translational criteria, including extraction yield, purity, structural preservation, scalability, solvent burden, reproducibility, environmental sustainability, and food-grade compatibility. Conventional alkaline-acid extraction remains widely utilized primarily because it delivers high extraction yields and reliable purity by effectively breaking down protein matrices. However, its massive chemical consumption imposes a heavy solvent burden and a severe environmental footprint. Methodologically, the extreme pH shifts inherent to this technique frequently induce extensive decarboxylation, causing irreversible structural modification or degradation of the underlying polymer network, which can directly compromise its final antioxidant and antimicrobial functionalities (Pralea et al., 2019). Furthermore, the risk of chemical contamination and toxic chemical poisoning introduces a substantial barrier to its food-grade application.

In contrast, green extraction methods like enzyme-assisted and ILs protocols offer excellent structural preservation, effectively maintaining the native morphology and functional integrity of isolated melanosomes (Pralea et al., 2019). While these methods offer high purity, moderate-to-high yields, and reduced environmental footprints through reusable solvent systems, they face major limitations in scalability and industrial reproducibility due to long incubation times, complex downstream processing, and high cost-prohibitive operational parameters.

Water-based extraction represents the safest and most ecologically benign method, providing high food-grade potential and scalability. However, it is fundamentally constrained by moderate yields and purity, as water alone is insufficient to degrade the complex carbohydrate and protein barriers shielding natural melanin granules (Thaira et al., 2019).

Table 3 summarizes the comparison among different methods of melanin extraction in terms of yield, structure, uses, and environmental effects. These limitations highlight the gap between laboratory-scale success and industrial application. Future research should focus on developing hybrid extraction strategies that combine the strengths of existing methods while improving scalability, sustainability, and cost-effectiveness.

**Table 3:** Comparison method of melanin extraction.

**Extraction method**	**Yield**	**Purity**	**Structural preservation**	**Scalability**	**Environmental impact**	**Food grade potential**	**Author(s)**
Alkaline-acid	High	High	Moderate possibility of structural modification due to harsh pH	High	High consumption of chemical	Moderate potential of chemical poisoning	Liu et al. (2003)
Enzyme-assisted	Moderate	High	High	Moderate	Low	High	Xie et al. (2021)
Ionic-liquids	High	High	Very high	Not available	Low chemical can be reused	Promising	Luo, Liu & Chen (2023)
Water	Moderate	Moderate	High	High	Low	High	Xie et al. (2021)

As these diverse extraction strategies yield materials with vastly different chemical behaviors, evaluating their subsequent food applications requires a precise categorization framework. Cephalopod-derived materials can be divided into four distinct processing tiers, including (1) whole squid ink, which is the raw, unfractionated fluid containing the polymer suspended alongside native proteins, amino acids, and minerals; (2) crude melanin extracts, where minimal mechanical processing leaves the protective structural protein matrix intact; (3) melanin-enriched fractions, which undergo preliminary isolation to reduce free lipids and non-pigment cellular elements; and (4) purified melanin, which represents the isolated biopolymer entirely stripped of accompanying matrix components. This classification is essential because the functional, sensory, or preservative outcomes observed in food applications are frequently driven by the collective, synergistic action of the complex ink matrix rather than the isolated polymer alone.

## Melanins and squid-derived materials application in the food industry

Over the past decades, the food industry has expanded rapidly to meet increasing population demands and evolving consumer preferences. Modern consumers are more aware of nutritional quality and health implications, which has intensified the demand for safe, functional, and sustainably sourced food ingredients (Galanakis, 2024). Table 4 summarizes experimentally validated applications of cephalopod-derived materials across varying processing tiers in food systems, highlighting their sources, incorporation methods, and reported functional outcomes. Across studies, materials ranging from unfractionated whole ink to crude extracts and purified isolates have been incorporated into food formulations, edible coatings, and polymer-based packaging materials. Reported laboratory findings suggest potential improvements in antioxidant activity, oxidative stability, and antimicrobial performance. While these preliminary studies suggest that these marine-derived matrices may contribute to food preservation, current evidence remains largely confined to model systems and laboratory-scale investigations. In addition, some studies reported modifications in physicochemical properties such as UV protection, barrier performance, and mechanical reinforcement in food packaging systems. These preliminary outcomes suggest the functional potential of melanin as a multifunctional food-grade ingredient beyond its traditional role as a pigment, though industrial scale-up remains validated.

**Table 4:** Applications of cephalopod-derived materials across varying processing tiers in food systems.

**Application**	**Melanin source**	**Purification status**	**Characterization technique**	**Food system**	**Incorporation method**	**Functional outcome**	**Attributable to melanin**	**Author(s)**
Natural colorant	Squid ink (*Todarodes pacificus*)	Water-soluble ink extract	UV-Vis, FTIR, SEM	Beverage and model food systems	Water-soluble ink extract	Model system	Partial	Liu et al. (2023)
Active packaging	Commercial cuttlefish ink	Whole ink	SEM, TEM, FTIR	Pectin/biopolymer film systems	Direct incorporation into polymer matrix with addition of Cu^2+^	*In vitro* film evaluation	No	Liang et al. (2023a)
Active packaging	Commercial cuttlefish ink	Whole ink	SEM, TEM, XRD	Meat products under packaging system	Incorporated into nanocomposite packaging film	Lab-scale food matrix	N/A	Liang et al. (2023b)
Active packaging	Sepia ink	Ink extract	FESEM	Chitosan/agar/ gelatine films	Direct blending of ink extract into polymer matrix	*In vitro* film evaluation	N/A	Roy & Rhim (2019)
Natural preservative	Squid ink (*Loligo duvacelii*)	Crude melanin-rich extract	Not reported	*In vitro* foodborne pathogen assays	Crude melanin-rich extract	*In vitro* screening	Yes	Vijaya Lakshmi et al. (2024)
Nutraceutical	Cuttlefish ink (*Sepia pharaonis*)	Purified melanin	SEM, FTIR	Cell-based model (Clone-9 hepatocytes)	Purified melanin extract applied *in vitro*	*In vitro* cell culture	Yes	Le et al. (2024)
Functional food	Squid ink (*Loligo* sp.)	Whole ink	N/A	Bakery product	Direct incorporation of squid ink into pizza dough formulation	Food matrix evaluation	No	Sidika, Andriani & Fikri (2022)
Flavor enhancer	Squid ink (*Loligo sp.*)	Whole ink	N/A	Seasoning paste	Developed as squid ink–based seasoning paste added during cooking	Food matrix evaluation	No	Rahmadi, Ahmad & Maqfira (2024)
Flavor enhancer	Squid ink (*Loligo vulgaris*)	Whole ink	N/A	Tortilla chips	Direct incorporation into formulation	Food matrix evaluation	No	Mulyani et al. (2019)

**Notes.**

N/A, Not available.

FESEM Field Emission Scanning Electron Microscopy FTIR Fourier-Transform Infrared spectroscopy UV-Vis Ultraviolet-Visible spectroscopy SEM Scanning Electron Microscopy TEM Transmission Electron Microscopy XRD X-ray Diffraction

### Black food colorant

Food colorants derived from both natural and synthetic sources are widely used to enhance the visual appeal of food products and influence consumer perception. Although synthetic colorants remain dominant, concerns regarding their safety, including allergic reactions, gastrointestinal disturbances, and potential neurobehavioral effects, have driven increasing interest in natural alternatives (Ma et al., 2025; Parlak et al., 2024). However, natural pigments often exhibit limitations such as low thermal stability, sensitivity to light, undesirable flavors or aromas, and relatively low extraction yields that require higher raw material input (Thomsen, Nielsen & Borodina, 2024).

Among natural pigments, compounds such as anthocyanins, betalains, carotenoids, and chlorophylls are commonly used, each with specific functional and stability constraints (Ghosh et al., 2022). In this context, melanin, a natural black pigment, has emerged as a promising alternative in model food systems due to its superior stability under heat and light conditions (Wu et al., 2018). Melanin, particularly when utilized as a component of cephalopod ink, has been traditionally used in various cuisines to impart black coloration to foods such as pasta, rice dishes, and baked products (Samanta, Sarkar & Chakraborty, 2024). This historical use highlights its potential for broader application in commercial food systems, although systematic validation under industrial processing conditions remains limited.

### Nutraceutical food ingredients

Food ingredients rich in antioxidant compounds play a crucial role in prolonging shelf-life and preserving food quality by inhibiting lipid oxidation and oxidative degradation (Araújo et al., 2026). ROS generated during processing and storage can accelerate food deterioration, and antioxidant compounds help mitigate these effects (Dos Santos et al., 2025). In this context, cephalopod-derived materials, ranging from whole ink to crude preparations, have gained attention due to their strong radical scavenging properties demonstrated primarily in chemical model systems.

Melanin extracts derived from natural sources have indicated exploratory *in vitro* antioxidant capacity, including high DPPH radical scavenging activity, which in some chemical assays is comparable to that of ascorbic acid at similar concentrations (Mustakim et al., 2024). While these spectrophotometric chemical assays suggest a strong inherent antioxidant potential, they do not automatically guarantee equivalent efficacy in complex food matrices or *in vivo* systems. The application of whole squid ink in food formulations has been primarily explored in bakery and dough-based products. For instance, unfractionated squid ink has been incorporated into products such as pizza and buns to enhance visual properties while maintaining acceptable sensory quality (Sidika, Andriani & Fikri, 2022; Wicaksono & Lastariwati, 2023). A study using the 4D model (define, design, develop, disseminate) reported that the incorporation of 10% whole squid ink produced buns with acceptable sweet and savory flavor profiles and soft texture. In addition, while higher ink incorporation levels have been associated with increased antioxidant activity in raw formulations (Kumar et al., 2024), direct testing on the final baked pizza matrix showed that the antioxidant capacity became practically inactive (IC_50_ >  200 ppm), likely due to structural and thermal degradation during high-temperature oven baking (Sidika, Andriani & Fikri, 2022). This highlights that the observed technological effects are heavily dependent on the processing state of the whole ink matrix rather than being directly attributable to the isolated melanin polymer alone.

### Flavor enhancer and additive

Cephalopod ink has been widely used as a natural flavor enhancer in various culinary applications, such as *ikasumi jiru* (Japanese soup), *chipirones en su tinta* (squid in ink sauce), *cavianne* (caviar imitation), and *arroz negro* (rice cooked with squid ink and cuttlefish) (Le et al., 2024). This characteristic umami taste is largely attributed to the presence of free amino acids, particularly glutamic acid, similar to other seafood such as oysters, mussels, shrimps, and scallops (Salleh et al., 2023). Since raw cephalopod ink is a complex biological matrix, these flavor-enhancing effects are tied to its overall chemical composition rather than the isolated melanin polymer itself.

Squid ink has also been incorporated into food products as a functional seasoning ingredient. For example, a squid ink-based seasoning paste **was** developed for fried rice, which resulted in a pronounced umami flavor without an undesired fishy off-note (Rahmadi, Ahmad & Maqfira, 2024). In bakery and snack applications, unfractionated squid ink has been added to cupcakes and tortilla chips, where sensory evaluations showed improved consumer scores for taste, aroma, and appearance. In particular, low concentrations (*e.g.*, 1%) in tortilla chips were found to be most acceptable to panelists (Vioni et al., 2018; Lestari et al., 2019). These sensory and formulation outcomes confirm that culinary acceptability depends entirely on the whole-ink organic matrix, as purified melanin isolates possess no inherent flavor-enhancing properties.

### Natural preservative

Preservatives are food additives used to extend shelf-life by inhibiting microbial growth and reducing lipid oxidation (Dong et al., 2024). Although synthetic preservatives such as benzoates, sorbates, nitrates, and formaldehyde are highly effective, concerns over potential adverse health effects, including allergic reactions and respiratory complications, have increased interest in natural alternatives (Hugo & Hugo, 2015). In response to growing demand for clean-label ingredients, natural compounds such as carnosic acid, vitamin E, and rosmarinic acid have been explored for their antimicrobial and antioxidant properties (Singh et al., 2025b). Similarly, whole cephalopod ink and its crude extracts have shown promising preservative potential in preliminary food trials. However, these preservative and antimicrobial outcomes are primarily whole-matrix effects driven by endogenous bioactive peptides and lysozymes within the raw ink, rather than being directly attributable to the isolated melanin polymer itself.

Studies have reported that food products treated with whole squid ink or crude preparations exhibit reduced lipid oxidation, lower histamine levels, and decreased microbial loads. For example, fish products treated with unfractionated squid ink, such as smoked sardines and canned seabream, showed improved quality parameters, including lower total viable counts and decreased total volatile base nitrogen (Martinez et al., 2024; Essid et al., 2022). In addition, crude extracts from squid ink have been applied to meatballs and noodles, extending shelf-life under ambient conditions to approximately 30 h (Aulia et al., 2013). Because these studies utilize complex, unpurified matrices, attributing the shelf-life extension solely to the melanin fraction oversimplifies the chemical interactions involved. Furthermore, benchmarking these crude whole-ink effects against standard commercial synthetic preservatives remains a critical gap in the current literature.

### Edible film and coating

Edible films and coatings are thin, consumable layers formed from hydrocolloids and lipids applied onto food surfaces such as fruits, vegetables, cheese, and meat *via* dipping, spraying, or brushing to maintain quality and extend shelf-life. Ideally, an edible coating should exhibit adequate transparency, effective barrier properties, and minimal impact on baseline sensory attributes, including taste, odor, and appearance (Yadav, Pal & Gaba, In press; Karnwal et al., 2025). In this context, crude melanin extracts and whole-ink fractions have emerged as experimental functional additives for biopolymer coating systems.

In addition to its extraction from cephalopod ink glands, crude melanin-containing fractions can be obtained from shellfish by-products such as mussel shells. Ke et al. (2024) reported that a crude melanin extract obtained from *Mytilus edulis* shells and incorporated into edible coatings significantly delayed lipid oxidation in fish fillets during cold storage, preserving quality parameters for up to eight days. This protective effect is attributed to the radical-scavenging ability of melanin, particularly its theoretical capacity to neutralize alkyl peroxyl radicals (ROO−), generated during lipid oxidation initiated by hydroperoxyl radicals (HOO−) (Mavridi-Printezi et al., 2023). Nevertheless, the dark coloration imparted by melanin presents a distinct visual modification that may influence consumer acceptability which requires rigorous sensory optimization.

### Food packaging

Plastic, glass, metal, and paper are commonly used food packaging materials. Plastic remains dominant due to its durability, low cost, and favorable mechanical properties (Gaspar & Braga, 2023). However, increasing plastic production has raised environmental concerns, particularly related to microplastic contamination and food safety risks (Najahi et al., 2025; Chinglenthoiba et al., 2025). To address these issues, biodegradable and active packaging systems have been actively investigated. Crude melanin-containing extracts has been incorporated into polymer matrices such as pectin, gelatin, carrageenan, and polyvinyl alcohol due to its core antioxidant, UV-absorbing, and antimicrobial properties (Liang et al., 2023a; Li et al., 2025).

Extract-functionalized packaging systems have suggested baseline food quality preservation under restricted laboratory conditions by slowing oxidation and microbial growth, thereby extending shelf-life (Shankar, Wang & Rhim, 2019; Liang et al., 2023b; Kumar et al., 2025). In addition, the crude extract combined with metal ions such as Cu^2^^+^ has been used to mimic antioxidant enzyme systems, altering the functional performance of the underlying packaging materials. For example, cherry tomatoes stored in melanin-functionalized packaging showed reduced spoilage and maintained visual quality after 15 days of storage (Liang et al., 2023a). In another study, Liang et al. (2023b) showed that the addition of melanin to pectin films enhanced thermal stability, tensile strength and water vapor barrier performance. Chicken, beef, and pork packed with these nanocomposite films exhibited a decreasing trend in total viable counts, indicating localized and exploratory antibacterial activity at a benchtop scale. Despite these promising outcomes, current evidence supporting melanin incorporation into food packaging matrices is at an early, exploratory stage of development. Most peer-reviewed studies report the development of packaging materials exclusively at a benchtop laboratory scale, without comparative benchmarking against commercially available active packaging systems. Furthermore, critical translation parameters such as cost-effectiveness, large-scale processing stability, migration kinetics of melanin components into food fat/aqueous simulants, long-term toxicity profiles, and regulatory approval pathways have yet to be holistically resolved. Therefore, extensive validation in real-world storage conditions and industrial scale-up trials is required before melanin-based food packaging technologies can be considered commercially viable.

## Current Limitations and Future Commercial Opportunities

Despite its promising properties in laboratory settings, translating natural marine melanin to commercial food applications is hindered by fundamental structural, chemical, and methodological bottlenecks. Structurally, natural nanomaterials such as melanin from squid ink face structural inconsistency, variable composition, and fluctuating bioactivity due to differences in squid species, diverse natural surroundings, and localized dietary conditions based on individual geographical locality (Xia et al., 2026). This includes complex structural heterogeneity where eumelanin and pheomelanin coexist as a mixed polymer known as a casing model, where eumelanin acts as an outer casing for pheomelanin (Pralea et al., 2019). This coexistence can alter or completely compromise the core photoprotective and antioxidant functionalities of eumelanin, as the inclusion of pheomelanin alters the overall performance (Derby, 2014; Morgan, Lo & Fisher, 2013). In terms of radiation blocking, eumelanin has the ability to convert UV radiation into harmless heat, while pheomelanin traps the UV radiation and transfers it to surrounding molecules, introducing highly inconsistent functional outcomes in food matrices (Pattnaik et al., 2024).

Methodologically, current extraction and isolation protocols face severe standardization and reproducibility deficits across the literature. In defining the purity of melanin isolated from squid ink, studies explain that purification of naturally synthesized melanin is exceptionally difficult because the molecule itself is tightly bound with carbohydrates, proteins, and lipids in its original biological matrix (Solano, 2014). Consequently, most common isolation routines rely on aggressive chemical treatments using strong acids, bases, or a combination of both (d’Ischia et al., 2015). This harsh processing frequently leads to over-decarboxylation and irreversible extraction-induced structural modification of the underlying melanin network. Furthermore, standard analytical verification techniques to confirm purity heavily rely on identifying a simple UV spectrophotometric absorption peak between 300 nm and 335 nm, a method that fails to capture structural degradation or subtle changes within the polymer matrix (Shin, 2026). Due to these differences and limited direct comparison among studies, the standardization of melanin nanoparticles remains highly complicated. To minimize batch variability for viable industrial application, controlled aquaculture systems operating under Good Manufacturing Practice (GMP) standards must be further developed.

Navigating the complex global regulatory framework remains a critical barrier to commercialization. A lack of systematic regulatory criteria, alongside limited empirical data regarding the long-term toxicity, bioaccumulation, or potential human oral intoxication of these novel isolated fractions, has significantly slowed regulatory approval for direct food-contact and food additive applications. While crude cephalopod ink possesses a long history of traditional culinary use, utilizing isolated or highly purified melanin fractions demands exhaustive toxicological vetting. Comprehensive, standardized human risk assessments, *in vivo* profiling, and clear safety benchmarking are still critically required to satisfy stringent food regulatory parameters and overcome consumer scepticisms.

## Conclusion

Marine-derived melanin represents a highly versatile biomolecule possessing documented antioxidant, antimicrobial, and UV-shielding properties that offer substantial promise for the food sector. However, its immediate transition from laboratory-scale proof-of-concepts to industrial food applications faces deep chemical and methodological bottlenecks. Much of the historical literature fails to rigorously differentiate between the activities of truly purified melanin polymers and multicomponent matrices like crude ink extracts or partially purified fractions, making definitive functional attribution complex. Furthermore, classical extraction techniques often induce structural degradation and batch-to-batch heterogeneity, which undermines product standardization. While green extraction methods show immense environmental promise, their extraction yields and economic viability still require optimization before widespread industrial implementation can be realized.

## Future Research Directions

To bridge the gap between laboratory exploratory formulations and real-world industrial implementation, the upstream manufacturing pipeline must prioritize scalable, reproducible, and standardized green extraction techniques. Emerging green methodologies including microwave-assisted extraction, high-pressure processing, ultrasound, supercritical CO_2_, pulsed electric fields, and radiofrequency show strong potential for efficiently isolating bioactive compounds while reducing environmental impacts and solvent burdens. Optimization and hybrid integration of these non-thermal and thermal approaches can significantly overcome the limitations of conventional alkaline–acid methods, particularly by minimizing extraction-induced degradation, suppressing structural network alterations, and correcting batch-to-batch consistency issues. Resolving these upstream extraction variations is a critical prerequisite because a reliable, structurally unified polymer supply is necessary to ensure predictable functional behavior when incorporated into downstream food applications.

In addition, future research must pivot downstream by shifting focus away from simplified, chemical-based spectrophotometric assays (*e.g.*, DPPH or ABTS) toward rigorous validation within highly complex, real food matrices. Understanding the precise chemical interactions of marine melanin with key native food components, such as proteins, lipids, and carbohydrates, is essential to predict its true performance and sensory stability during commercial thermal processing and retail storage. Correspondingly, future investigations must transition from isolated model tests to comprehensive comparative benchmarking against established commercial standards when evaluating melanin-functionalized active films and edible coatings. This active food-system verification is mandatory to establish actual technological readiness and to ensure that the material’s strong *in vitro* radical scavenging properties successfully translate into a real-world shelf-life extension.

Finally, achieving proven technological efficacy within real food matrices must be coupled with rigorous verification of material safety, migration kinetics, and global regulatory compliance. As pure isolated chemical fractions behave differently than traditional whole foods, the lack of systematic empirical data regarding long-term oral toxicity, bioaccumulation, and direct consumer safety remains a primary barrier to commercialization. Exhaustive *in vivo* toxicological profiling and explicit assessment of migration behaviors within polymeric packaging systems is crucial to satisfy strict regulatory frameworks, such as those governed by the FDA and EFSA. Concurrently, conducting comprehensive techno-economic analyses and life-cycle assessments will be required to confirm that the multiple processing and washing cycles involved in manufacturing remain economically viable.

## Supplemental Information

10.7717/peerj.21855/supp-1 Supplemental Information 1 The uses of animal melanin in various food industries

## Additional Information and Declarations

### Data Availability

The following information was supplied regarding data availability: This is a Literature Review.
